# Potential Therapeutic Targets in Obesity, Sleep Apnea, Diabetes, and Fatty Liver Disease

**DOI:** 10.3390/jcm13082231

**Published:** 2024-04-12

**Authors:** Christina Gu, Nicole Bernstein, Nikita Mittal, Soumya Kurnool, Hannah Schwartz, Rohit Loomba, Atul Malhotra

**Affiliations:** 1 Department of Medicine, University of California San Diego, 9500 Gilman Drive, La Jolla, CA 92037, USA; nbernstein@health.ucsd.edu (N.B.); nmittal@health.ucsd.edu (N.M.); skurnool@health.ucsd.edu (S.K.); roloomba@health.ucsd.edu (R.L.); 2 Weill Cornell Medicine, 1300 York Ave, New York, NY 10065, USA; has4021@med.cornell.edu

**Keywords:** obesity, metabolic syndrome, MASLD, NAFLD, hepatic steatosis, OSA, CPAP, diabetes, GLP-1, GIP, glucagon, bariatric surgery

## Abstract

Obesity and metabolic syndrome affect the majority of the US population. Patients with obesity are at increased risk of developing type 2 diabetes (T2DM), obstructive sleep apnea (OSA), and metabolic dysfunction-associated steatotic liver disease (MASLD), each of which carry the risk of further complications if left untreated and lead to adverse outcomes. The rising prevalence of obesity and its comorbidities has led to increased mortality, decreased quality of life, and rising healthcare expenditures. This phenomenon has resulted in the intensive investigation of exciting therapies for obesity over the past decade, including more treatments that are still in the pipeline. In our present report, we aim to solidify the relationships among obesity, T2DM, OSA, and MASLD through a comprehensive review of current research. We also provide an overview of the surgical and pharmacologic treatment classes that target these relationships, namely bariatric surgery, the glucagon-like peptide-1 (GLP-1), glucose-dependent insulinotropic polypeptide (GIP), and glucagon receptor agonists.

## 1. Introduction

Obesity has been increasingly pervasive over the past several decades, although the mechanism of the rising prevalence remains unclear [1]. Current estimates suggest that just over one-third of the US population is considered to have a healthy weight (defined by BMI 18.5–24.9 kg/m^2^), one-third fall in the overweight category (BMI 25–29.9 kg/m^2^), and one-third is obese (BMI > 30kg/m^2^) [2]. Current projections suggest one in two Americans will meet criteria for obesity by 2030. The mechanisms underlying obesity and the metabolic syndrome are intricate and complex. Obstructive sleep apnea (OSA), type 2 diabetes (T2DM), and metabolic dysfunction-associated steatotic liver disease (MASLD) are common comorbidities of obesity. While T2DM affects roughly 10% of the US population (www.cdc.gov/diabetes, accessed on 22 January 2024), it is estimated that OSA affects nearly 1 billion people worldwide [3]. It is estimated that about 25% of the U.S. population has non-alcoholic-associated fatty liver disease (NAFLD), recently renamed to MASLD, and about 20% of those with NAFLD have non-alcoholic steatohepatitis (NASH). As early as 2018, the leading cause of liver transplantation in U.S. women was shown to be NASH [4]. The rising prevalence of obesity and the resultant pervasiveness of its comorbidities, namely T2DM, OSA, and MASLD, hold critical adverse implications in healthcare, including rising mortality risk, impaired quality of life, and increased healthcare spending [2]. The complications from these disease states themselves also give rise to further problems, including cardiovascular, microvascular, skeletal, gastrointestinal, and neurocognitive diseases. The close association among these disease states begs the questions: What are the shared mechanisms underlying obesity and its comorbidities? What current US Food and Drug Administration-approved therapies might be beneficial for comorbid conditions? Looking ahead, what future therapies on the horizon might also target these mechanisms? Current literature has established connections between various permutations of the metabolic syndrome comorbidities (obesity, T2DM, OSA, and MASLD); however, the complex nature of the physiological axes connecting these diseases is still under investigation (Figure S1). Thus far, the current literature has yet to include a comprehensive review of T2DM, OSA, and MASLD in the context of obesity. Notably, OSA is also an underrecognized independent risk factor for the development of both T2DM and MASLD.

In this report, we first aim to discuss the current understanding of the interplay among obesity, T2DM, OSA, and MASLD. Next, we describe current therapeutic approaches to the metabolic syndrome axis and discuss how various treatment classes, including GLP-1 receptor agonists and triple G triagonists (combined glucagon, glucagon-like peptide-1, and glucose-dependent insulinotropic polypeptide receptor agonists), may transform our understanding of the physiological interconnectedness of these diseases. Finally, we draw attention to the potential therapeutic benefit of continuous positive airway pressure (CPAP) on disease states beyond OSA. The overarching aim of our review is to highlight the interconnectedness of T2DM, OSA, and MASLD and to provide an overview for the direct and indirect effects of obesity treatment on all of these diseases.

## 2. Relationship between OSA and T2DM

Sleep disturbances have commonly been associated with the development of obesity in epidemiological studies [5,6]. This finding could be explained by physiological studies that have shown suppression of leptin and increases in ghrelin with sleep deprivation, both of which are changes predicted to stimulate appetite and promote obesity [7]. It is unsurprising then, that short sleep has also been associated with incident diabetes mellitus, as well as the worsening of markers of insulin regulation [6,8,9,10]. OSA itself has been established as an independent risk factor for several metabolic and cardiovascular disease states, including hypertension, insulin resistance, fatty liver disease, atherosclerosis, and dyslipidemia. Treatment for sleep apnea can lead to improvements in blood pressure, although the impact of OSA treatment on glycemic control is less clear [11,12].

Multiple pathways are theorized to explain a causative effect of intermittent hypoxemia and sleep fragmentation on the development of insulin resistance and glucose dysregulation. One such pathway is via increased sympathetic neural activity. Laboratory assessments of patients with untreated OSA have demonstrated both increased sympathetic hormonal levels and activity that persists in the daytime and is reduced by consistent CPAP therapy [13,14]. Most processes involved in glucose control, including pancreatic insulin secretion and hepatic glucose production, are inhibited by elevated sympathetic tone [15]. In addition, cholinergic activity is directly linked to the secretion of incretin hormones, such as glucagon-like peptide-1 and glucose-dependent insulinotropic polypeptide. These incretin hormones act to augment insulin release. These findings suggest that patients with OSA are more predisposed to developing sympathetic hyperactivity and parasympathetic withdrawal, which collectively mediate glucose intolerance and thus T2DM [16]. Chronic intermittent hypoxia has been proposed to cause glucose intolerance through other proposed pathways, including the development of oxidative stress, systemic inflammation, activation of the hypothalamic–pituitary–adrenal (HPA) axis, pancreatic beta-cell apoptosis, and the alteration of adipokines, each of which leads to downstream effects on beta cell dysfunction and insulin resistance.

T2DM is also proposed to contribute to worsening OSA. Some observational evidences suggest that patients with diabetes and autonomic neuropathy demonstrate altered control of respiration and upper airway reflexes that promote airway patency, as seen in sleep-disordered breathing patterns among patients with diabetes [17,18]. This reverse causative relationship is also supported by a high prevalence of OSA among patients who are younger and non-obese with type 1 diabetes [19].

Unfortunately, a major subgroup of patients with T2DM are undertreated for OSA [20,21,22]. The Sleep-Ahead study showed that 87% of patients with T2DM and obesity also had clinically important OSA. After these patients and their providers received the diagnosis of OSA, over 95% of these patients with OSA remained untreated a year later [21,22,23]. Despite mixed results in studies assessing the impact of CPAP on glycemic control [11,24], the importance of addressing OSA in T2DM patients to reduce cardiometabolic risk is emphasized. Amongst patients with T2DM, there is a need for increased awareness to promote OSA as a valuable therapeutic target with implications on cardiometabolic health.

## 3. Relationship between OSA and MASLD

Hypoxia has been shown to accelerate the development of MASLD. One study using mouse models [25] demonstrated that hypoxia contributed to liver abnormalities only in the presence of obesity. Mice with diet-induced obesity were subject to chronic intermittent hypoxia; compared to lean mice that received the same hypoxic conditions, the obese mice exhibited markedly increased serum AST, ALT, and alkaline phosphatase levels and fasting glucose from baseline. In addition, the obese mice exhibited significantly higher levels of hepatic steatosis and inflammation on histology. Similar findings were reproduced in human studies. A study by Polotsky et al. [26] focused on patients presenting for bariatric surgery found that those with severe nocturnal hypoxemia tended to exhibit histologic signs of worse hepatic inflammation, including hepatocyte ballooning and perivesicular fibrosis, when compared to patients with mild sleep apnea. These findings are supported by other studies that observed nocturnal hypoxic episodes as a contributor to hyperlipidemia and steatohepatitis and as a possible risk factor for MASLD [27,28].

Considerable data from rodent studies have suggested that hypoxic effects on lipid metabolism may be responsible for the development of MASLD. In gene expression analysis studies, chronic intermittent hypoxia has been demonstrated to cause the upregulation of multiple genes responsible for the biosynthesis of cholesterol, triglycerides, fatty acid, and phospholipids [29]. It is not surprising then that increased enzymatic activity in hepatocytes would lead to increased lipid accumulation and hepatic steatosis. Other proposed indirect mechanisms include pancreatic beta-cell apoptosis and overactivation of the sympathetic nervous system, both of which lead to insulin resistance and thus predispose individuals to MASLD.

To date, most studies that have investigated the link between MASLD and OSA have focused on the increased risk of developing liver injury and inflammation among individuals with OSA. However, few have examined whether the inverse is true. One such study [30] reported that among patients with biopsy-proven MASLD, those with concurrent hepatic fibrosis had higher overall rates of OSA, compared to those without fibrosis. Another study demonstrated that the apnea–hypopnea index was significantly higher among those with moderate to severe MASLD, compared to those without MASLD [31,32,33]. The study by Chung et al. [34], which used a surrogate fatty liver index (FLI) score to identify MASLD, found that the risk for receiving an OSA diagnosis increased in a dose-dependent manner as FLI increased. This relationship remained consistent regardless of BMI and the presence of abdominal obesity and offers FLI as a potential tool used to identify individuals at high risk of OSA. These studies, although not necessarily suggestive of a causative effect by MASLD upon the development of OSA, do again underscore a close association between the two.

## 4. Relationship between T2DM and MASLD

Insulin resistance is believed to play a pivotal role in the development of fatty liver disease. On a cellular level, insulin signaling is initiated through the binding and activation of its cell-surface receptor, which triggers a cascade of phosphorylation and dephosphorylation events that ultimately lead to the translocation of glucose transporters into the cell membrane. The transporters then facilitate glucose influx down a concentration gradient from the extracellular space into the cytoplasm. Defects at any step of this cascade will result in issues with glucose uptake into cells and abnormalities with insulin sensitivity. Features seen in patients with obesity and diabetes, such as hyperglycemia, hyperinsulinemia, and the presence of free fatty acids, have been implicated in altering insulin signaling. Once peripheral insulin resistance has been established, hyperinsulinemia subsequently leads to increased fatty acid delivery into the liver, leading to increased hepatic triglyceride production and hepatic steatosis. Hepatic steatosis itself has been shown to lead to hepatic insulin resistance, which may contribute to the overall worsening of peripheral insulin resistance. Additionally, high levels of free fatty acids and hyperinsulinemia in the body have also been shown to lead to the production of free radicals, resulting in oxidative stress and an inflammatory response, including cytokine production, which are believed to further promote both insulin resistance and steatohepatitis. These latter mechanisms underlying the development of insulin resistance that have been reproduced in multiple molecular studies do suggest that the relationship between T2DM and MASLD may be bidirectional, or perhaps cyclic, in nature [35].

In clinical studies, the close association of T2DM and MASLD has been clearly illustrated. In a national survey of middle-aged patients with and without T2DM, it was found that the rate of steatosis was significantly higher in those with overweightness and obesity with T2DM versus without T2DM. T2DM was also found to significantly increase the proportion of those at moderate-to-high risk of fibrosis by two-fold [36], suggesting that T2DM may be predictive of the development of fibrosis in MASLD. Another study by Sung et al. compared the effect of ultrasound-diagnosed MASLD on the risk of developing incident T2DM among 12,000 South Korean adults over the span of 5 years [37]. After adjusting for confounding factors, MASLD doubled the risk of developing T2DM. Other cohort studies have consistently shown that MASLD is predictive of T2DM, whether the diagnosis of MASLD is made by imaging or biopsy [38]. In addition, MASLD has also been shown to increase microvascular complications of T2DM, including chronic kidney disease and retinopathy.

The recognition of this shared elevated risk underscores the importance of vigilant monitoring in individuals with the dual burden of MASLD and T2DM. Due to the impact of insulin resistance on the pathophysiology of MASLD, potential pharmacologic treatments for MASLD have focused on hypoglycemic agents, including metformin, SGLT2-I, PPAR agonists, GLP-1 receptor agonists, and multi-agonists [39]. These diabetes drug classes have shown varying levels of benefit in reducing liver enzyme levels, liver fat content, and histologic features of inflammation and fibrosis.

## 5. Treatment Approaches

In the above review, we discussed current literature that inform our current understanding of how obesity, diabetes, sleep apnea, and fatty liver disease are inter-related. Here, we discuss the surgical and pharmacologic treatments that act upon these relationships to benefit patients with metabolic syndrome. We also expand on the potential efficacy of CPAP for patients with OSA and other concomitant metabolic diseases.

## 6. Overview of Bariatric Surgery

Bariatric surgery has been established as a highly effective option for patients with obesity to achieve sustained weight loss. Common, established procedures include sleeve gastrectomy (SG), Roux-en-Y gastric bypass (RYGB), gastric banding, and biliopancreatic diversion with duodenal switch [40]. A review of the literature suggests that among the different types of bariatric surgery, SG and RYGB have the highest efficacy for major weight reduction, with similar long-term results. A randomized controlled trial comparing the two methods, the SM-BOSS trial by Peterli et al. [41], evaluated adults with morbid obesity undergoing sleeve gastrectomy compared with gastric bypass over the course of 5 years. At the end of the study, the SG group lost 61% BMI, compared to the RYGB group, which lost 68%, a difference that was not statistically significant. Overall complications within the first 30 days occurred more often in the RYGB group than the SG group. However, the rates of developing severe complications in this study, such as severe GERD after SG and severe dumping syndrome after RYGB, which required further surgeries, were not statistically significant. Similar effects of bariatric surgery on weight loss were seen in other studies, which also noted improved comorbidities, including diabetes and hypertension [42,43].

There is clear evidence that bariatric surgery achieves meaningful remission rates of T2DM. Two meta-analyses reviewed the long-term effects of surgery on glycemic control over more than 2–5 years. These found that bariatric procedures were likely to achieve sustained weight loss, A1c lowering, overall blood glucose reduction, and, in many cases, diabetes remission. In addition, it was also shown that bariatric surgery had a significant reduction on the incidence of complications, as well as overall mortality, among patients who received at least 5 years of follow-up [44,45]. Another longer-term study was the Swedish Obese Subjects (SOS) trial, a 15-year prospective matched cohort study which observed a diabetes remission rate of 30% among those who had received bariatric surgery, with fewer incidences of micro- and macrovascular complications [46]. In fact, a recent study found that participants had higher rates of diabetes remission after bariatric surgery, compared to medical/lifestyle management, at up to 12 years of follow-up [47].

With regards to its effects on liver disease, several studies have also shown improvement in MASLD both after SG and RYGB. In a meta-analysis [48], patients undergoing RYGB achieved significant reductions in steatohepatitis and fibrosis, while patients undergoing LSG had a significant reduction in steatohepatitis only. Studies have shown mixed data regarding the superiority of one bariatric method in reversal of MASLD. Interestingly, there is a cohort of patients that appear to develop new or progressively worsening MASLD after bariatric surgery. In 2019, Lee et al. [49] showed that 12% of patients (95% CI, 5–20%) developed new or worsening MASLD after bariatric surgery. Further examination of those with worsening disease after surgery suggests that those who lose weight more rapidly may be more susceptible, which is possibly related to malnutrition or malabsorption. Of note, this meta-analysis included variations in bariatric surgery beyond SG and RYGB, such as gastric banding and gastroplasty. Although studies show an overall benefit of bariatric surgery on the amelioration of MASLD, there is a significant portion of patients that may develop new or worsening disease; this is an important clinical consideration prior to surgical intervention.

The prevalence of patients with OSA among patients undergoing bariatric surgery should not be understated; some screening studies estimate that as many as 72% of the bariatric surgery population has OSA [50,51]. Dramatic and sustainable weight loss, as seen in these surgeries, does see remarkable improvement and, in some cases, even the resolution of OSA. There is substantial evidence that weight reduction alleviates upper airway collapsibility and reduces upper airway resistance, thereby promoting increased oxygenation and the reduction of apneic episodes [52]. A large-scale UK national registry cohort study by Currie et al. [53] followed over four-thousand bariatric surgery cases over the span of nine years, including SG, RYGB, and gastric banding. SG and RYGB were associated with a 50% increased likelihood of OSA remission, compared with gastric banding, consistent with the greater degree of weight loss seen in both SG and RYGB surgeries. It is important to recognize, however, that even with weight reduction, the presence of obstructive sleep apnea is not always totally reversible, and its degree of resolution is highly variable [54]. In the UK registry cohort study, about half of the remaining cases at follow-up by the end of the study duration saw a complete resolution of OSA. Other studies have also illustrated that some patients may redevelop OSA, despite maintaining weight loss [54,55].

## 7. Overview of GLP-1 and GIP–GLP-1 Agonists

Incretins are peptide hormones that are released from the intestine and brainstem in response to nutrient consumption and act primarily to lower serum glucose and increase satiety. The backbone of medical therapies used to treat metabolic syndrome utilizes analogs of the main incretin hormones: glucagon-like peptide-1 (GLP-1) and glucose-dependent insulinotropic polypeptide (GIP). GLP-1 is a 30-amino-acid-long peptide chain produced by enteroendocrine L cells in the distal ileum and colon and by neurons in the nucleus of the solitary tract of the brainstem. It acts to stimulate insulin production in pancreatic beta cells in a glucose-dependent manner and decreases glucagon secretion from pancreatic alpha cells. GLP-1 additionally has extra-pancreatic effects through the direct suppression of the appetite center and the slowing of gastric emptying, thus increasing satiety and reducing food-seeking behavior. GIP is a 4-amino-acid peptide secreted by K cells in the duodenum and jejunum. This short peptide hormone is stimulated by glucose hyperosmolarity in the intestine to induce insulin secretion. Given these properties, GLP-1 and GIP agonists have been subjected to intensive pharmacologic investigation for the treatment of obesity and T2DM (Figure S2).

The current FDA-approved GLP-1 receptor agonists for treating both T2DM and obesity include once-daily liraglutide, weekly semaglutide, and weekly tirzepatide injections. Tirzepatide, a dual GIP–GLP-1 agonist, is the latest to be approved and has demonstrated significant results in weight reduction and glucose control when compared with its predecessors. The phase 3 SURMOUNT-1 trial examined the efficacy of tirzepatide on weight loss against a placebo over the span of 72 weeks [56]. All three doses of tirzepatide being studied (5, 10, and 15 mg) found a significantly greater weight reduction than with the placebo after as soon as 20 weeks. These results were greater than the mean placebo-adjusted weight reduction with liraglutide and semaglutide but with a similar safety profile. Weight reduction with tirzepatide was also accompanied by other cardiometabolic benefits, including reductions in blood pressure, waist circumference, fasting glucose, fasting cholesterol levels, and aspartate aminotransferase levels, when compared with the placebo. Similarly, the SURPASS-3 MRI study was another phase 3 trial that examined tirzepatide but with a specific focus on its effects on liver changes as measured by MRI; this demonstrated meaningful reductions in liver fat content, volume of visceral adipose tissue, and abdominal subcutaneous adipose tissue among patients with T2DM receiving 52 weeks of 10 mg or 15 mg tirzepatide when compared with those taking insulin degludec [57]. The impact of these medications on OSA is yet to be established and is a topic of ongoing investigation, particularly the efficacy of tirzepatide on OSA reversal [58].

## 8. Overview of Triple G Triagonists

Currently in the pharmacologic pipeline is a class of medications called triple G receptor agonists, or triagonists. These act via three receptors; the mechanisms of GIP and GLP-1 receptor agonists have been described above. The third “G” is glucagon, which is a 29-amino-acid peptide hormone produced by pancreatic alpha cells. Glucagon is known for its hyperglycemic effects via the liver and works in a feedback loop with GIP and GLP-1 to achieve glucose homeostasis. Additionally, glucagon is also a catabolic peptide, acting to increase lipolysis and thermogenesis. Some biochemical studies have shown that glucagon exerts its effects in the CNS by promoting satiety as well. Through the fine-tuning of the combined triple peptide analog, preclinical studies have demonstrated that the resultant triple G receptor agonist can promote glucagon’s catabolic effects without exacerbating hyperglycemia [59].

One such triple G triagonist, retatrutide, has shown promising results in the treatment of obesity, T2DM, and MASLD. Initial data from three studies were revealed at the 2023 ADA conference. The Triple-Hormone-Receptor Agonist Retatrutide for Obesity was a phase 2 trial which examined retatrutide at various doses and dose-escalation regimens in patients with obesity [60]. Jastreboff et al. found that over the course of 48 weeks, all participants on the two highest doses of 8mg and 12mg lost at least 5% of weight, and those who were on 12 mg lost 24% of body weight on average. Those on 12 mg had a mean reduction of 19.6 cm waist circumference. The overall safety profile was similar to other GLP-1 agonists previously approved for obesity treatment, with the most common side effect being adverse GI events. A sub-study from the trial recruited participants with obesity and MASLD and utilized MRI and liver injury biomarkers to track changes in hepatic steatosis. Findings at the ADA press release [61] revealed that those with MASLD had a normalization of fat levels in the liver after 48 weeks of treatment on the highest dose of retatrutide, suggesting that MASLD can be treatable and reversible. A third study by Rosenstock et al. examined the efficacy and safety profile of retatrutide for the treatment of T2DM [62]. Participants lowered their A1c by 1.3–2% after taking 4–12 mg for about six months, compared to no change with the placebo and a 1.4% reduction with dulaglutide.

Data regarding the effect of triple G agonists on OSA specifically are still sparse, although phase 3 trials for retatrutide are projected to enroll patients with OSA. Given the impacts of these drugs on weight loss, glycemic control, and fatty liver disease, it is reasoned that patients with OSA may still see benefit.

## 9. Implications of CPAP Therapy on T2DM and MASLD

As evidenced above, OSA has high prevalence and tightly co-exists with other metabolic diseases, including central obesity, dyslipidemia, diabetes, and fatty liver disease. The direct effects of CPAP therapy on these metabolic disease states, however, is still under investigation. Short-term, randomized control trials evaluating CPAP efficacy on glycemic control for patients with concurrent OSA and prediabetes have demonstrated overall improved insulin sensitivity. One such study by Pamidi et al. [63] randomized participants to receiving either 8 h of nightly CPAP or a placebo over 2 weeks. Although the study did not find a difference in fasting glucose between the two groups, it did demonstrate improvement in response to glucose tolerance tests and in measured fasting insulin levels. Norepinephrine levels were also markedly lower in those receiving CPAP, which again supports the theory that a reduction in sympathetic activity could mediate improved glycemic control. However, a meta-analysis of RCTs examining CPAP effects on T2DM over a longer period concluded that CPAP does not significantly improve A1c or fasting glucose [64]. CPAP effects on MASLD are also under investigation. As described above, there are robust literature that demonstrate chronic intermittent hypoxic episodes as a contributor to oxidative stress on the liver, accelerated progression to steatohepatitis, and increased inflammation and fibrosis [28,29,30,31]. Reversal of hypoxic insults to the liver with CPAP alone, however, has yielded mixed results to date. One study examined the effects of 6 months of CPAP treatment for patients with both MASLD and OSA [65]. When controlling for weight changes over the duration of the study, the authors found no significant difference between the placebo and CPAP use on intrahepatic triglyceride content as measured by MRI, on FibroScan results, or serum liver function tests. A few other studies have shown that liver enzymes may be elevated in those with OSA and may also be lowered with CPAP [66]; however, these studies provide only indirect data with regards to specific liver tissue effects from CPAP use. Given the previous research findings that have supported chronic intermittent hypoxia as an important risk factor for glucose intolerance and hepatic steatosis, it is unclear why the results of CPAP trials on patients with T2DM and MASLD have been largely negative. Considerations for future studies include changes in trial design to promote better CPAP adherence and more direct methods to measure T2DM and MASLD outcomes. Alternative treatments for OSA should also be considered in these trials to better reflect real-world clinical practice, such as the use of oral appliances or surgical treatments.

## 10. Future Directions

For both clinicians and researchers, a number of practical questions remain for our understanding of and management of OSA. First, the specific mechanisms underlying obesity-related sleep apnea are still not entirely clear. Abnormalities in pharyngeal anatomy and the tongue have been reported, as well as the impact of abdominal obesity on end-expiratory lung volume [67,68,69,70]. However, other abnormalities in control of breathing and upper airway dilator muscle function have been suggested as potential issues [71]. Second, the optimal therapy for OSA for patients with obesity has not yet been found. Ongoing studies will likely inform this discussion. However, it seems highly likely that treatments of both obesity and OSA will be necessary to optimize clinical outcomes in afflicted patients [72]. Third, it is not yet clear whether there are predictors of cardiometabolic outcomes in obese people with OSA that could be used to stratify risk. Robust biomarkers and predictive markers could be used to identify high-risk patients and potentially stratify interventional approaches for those patients most likely to benefit. Approaches including mass spectroscopy could be used to discover new biomarkers, which predict the risk of OSA and associated complications [73,74].

Additional questions remain for the management of patients with T2DM and MASLD as well. There are few U.S. Food and Drug Administration-approved drugs that exist to treat MASLD [75], and the mainstay of therapy continues to focus on lifestyle interventions targeting dietary changes and weight loss. Given what we know now, more work needs to be performed to establish standardized screening criteria that help recognize those at risk of developing MASLD who require more aggressive therapy, whether this is in the form of the early initiation of tirzepatide or referral to bariatric surgery. Similarly, given the role that insulin resistance and autonomic neuropathy might play on the development of MASLD and OSA, respectively, more defined screening tools for the recognition of liver and sleep abnormalities may be necessary for patients with diabetes. It is also unclear whether patients with mild OSA but concomitant T2DM or MASLD require a more aggressive push toward starting CPAP therapy. More prospective studies are needed to better inform these questions.

Finally, although not directly addressed in this review article, diet is a foundational pillar underlying metabolism that is still not fully understood. Changes in how and what we eat have been suggested to play a role in the development of metabolic syndrome. Many specific food components and nutrients, as well as various dietary patterns, are undergoing studies to test their therapeutic potential. Polyphenolic compounds, for example, which are naturally occurring in fruits, vegetables, and cereals, may be one group of metabolites utilized for treatment, given their antidiabetic and cardioprotective properties [54]. Dietary formulations, including the Mediterranean diet, which is rich in polyphenols, may also hold promise in reducing oxidative stress in sleep apnea.

## 11. Conclusions

Understanding the intricate connections between obesity, OSA, T2DM, and MASLD is paramount for developing effective therapeutic strategies. Sleep, often overlooked, plays a crucial role in the progression of these conditions. The ongoing research, as exemplified by the SURPASS-3 MRI study and triple G medication studies, offers promising avenues for addressing NAFLD and improving overall health outcomes. By integrating sleep health considerations into treatment approaches, there exists the potential for preventing and managing obesity, OSA, and T2DM, thereby enhancing the quality of life for individuals affected by these conditions.

## Data Availability

No new data were created or analyzed in this study. Data sharing is not applicable to this article.

## Supplementary Materials

The following supporting information can be downloaded at: https://www.mdpi.com/article/10.3390/jcm13082231/s1, Figure S1: An overview of the proposed mechanisms underlying MASLD, OSA, and T2DM and some of the current therapies that target these associations. Figure S2: An overview of GIP–GLP-1 effects on glucose and satiety.
