# Supplementary material for: Potential Therapeutic Targets in Obesity, Sleep Apnea, Diabetes, and Fatty Liver Disease
Source: J Clin Med. 2024 Apr 12;13(8):2231. doi: 10.3390/jcm13082231 (PMC11050527; doi:10.3390/jcm13082231)

**Supplementary Materials**

**Figure S1: An overview of the proposed mechanisms underlying MASLD, OSA, and T2DM and some of the current therapies that target these associations**

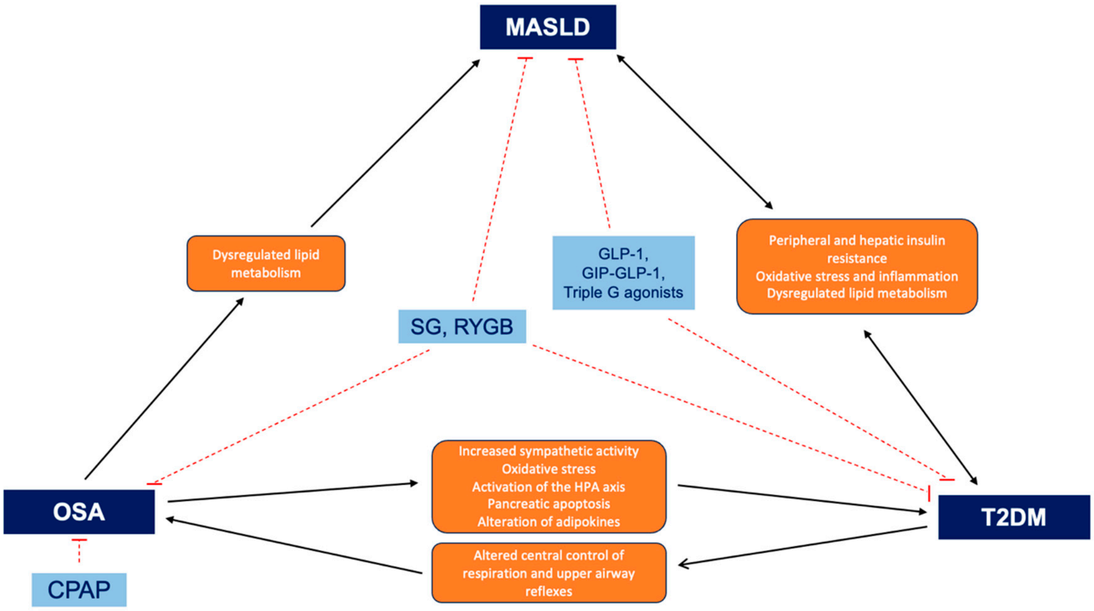


**Figure S2: An overview of GIP-GLP-1 effects on glucose and satiety**

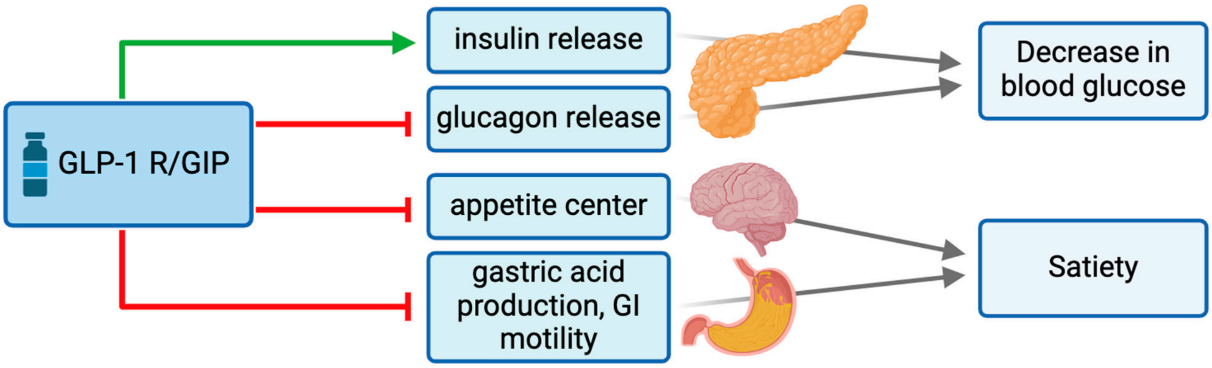

Supplement: Supplementary file 1 [file jcm-13-02231-s001.zip › jcm-2912940-supplementary.pdf]
